# Lung cancer screening with low-dose CT: definition of positive, indeterminate, and negative screen results. A nodule management recommendation from the European Society of Thoracic Imaging

**DOI:** 10.1007/s00330-025-11648-4

**Published:** 2025-07-01

**Authors:** Annemiek Snoeckx, Mario Silva, Helmut Prosch, Jürgen Biederer, Thomas Frauenfelder, Fergus Gleeson, Colin Jacobs, Hans-Ulrich Kauczor, Anagha P. Parkar, Cornelia Schaefer-Prokop, Mathias Prokop, Marie-Pierre Revel

**Affiliations:** 1 Department of Radiology, Antwerp University Hospital, Edegem, Belgium; 2 Faculty of Medicine and Health Sciences, University of Antwerp, Wilrijk, Belgium; 3 Scienze Radiologiche, Department of Medicine and Surgery (DiMeC), University of Parma, Parma, Italy; 4 Department of Biomedical Imaging and Image-Guided Therapy, Medical University of Vienna, Vienna General Hospital, Vienna, Austria; 5 Department of Diagnostic and Interventional Radiology, University Hospital of Heidelberg, Heidelberg, Germany; 6 Translational Lung Research Center Heidelberg (TLRC), Member of the German Lung Research Center (DZL), Heidelberg, Germany; 7 Faculty of Medicine, University of Latvia, Riga, Latvia; 8 Faculty of Medicine, Christian-Albrechts-Universität zu Kiel, Kiel, Germany; 9 Institute of Diagnostic and Interventional Radiology, University Hospital Zurich, University of Zurich, Zurich, Switzerland; 10 Department of Oncology, University of Oxford, Oxford, UK; 11 Department of Medical Imaging, Radboud University Center, Nijmegen, The Netherlands; 12 Department of Radiology, Haraldsplass Deaconess Hospital, Bergen, Norway; 13 Department of Clinical Medicine, Faculty of Medicine and Dentistry, University of Bergen, Bergen, Norway; 14 Department of Radiology, Meander Medical Center, Amersfoort, The Netherlands; 15 Department of Radiology, University Medical Center Groningen, Groningen, The Netherlands; 16 Department of Radiology, Cochin Hospital, Université Paris Cité, Paris, France

**Keywords:** Lung cancer, Pulmonary nodule, Screening programs (diagnostic), Artificial intelligence, Low-dose computed tomography

## Abstract

**Abstract:**

Early detection of lung cancer through low-dose CT lung cancer screening in a high-risk population has proven to reduce lung cancer-specific mortality. Nodule management plays a pivotal role in early detection and further diagnostic approaches. The European Society of Thoracic Imaging (ESTI) has established a nodule management recommendation to improve the handling of pulmonary nodules detected during screening. For solid nodules, the primary method for assessing the likelihood of malignancy is to monitor nodule growth using volumetry software. For subsolid nodules, the aggressiveness is determined by measuring the solid part. The ESTI-recommendation enhances existing protocols but puts a stronger focus on lesion aggressiveness. The main goals are to minimise the overall number of follow-up examinations while preventing the risk of a major stage shift and reducing the risk of overtreatment.

**Key Points:**

***Question*** *Assessment of nodule growth and management according to guidelines is essential in lung cancer screening*.

***Findings*** *Assessment of nodule aggressiveness defines follow-up in lung cancer screening*.

***Clinical relevance*** *The ESTI nodule management recommendation aims to reduce follow-up examinations while preventing major stage shift and overtreatment*.

## Introduction

Lung cancer is the leading cause of cancer death worldwide [1]. When diagnosed based on symptoms, most lung cancers are at an advanced stage and not eligible for complete surgical resection, which offers the greatest potential for long-term survival [2]. This is why screening reduces lung cancer mortality, by detecting lung cancer at an early, preclinical phase. This approach has recently been demonstrated to cause stage shift and improved survival in the US, following the introduction of lung cancer screening with low-dose computed tomography (LDCT) [3]. Lung cancer screening differs from other cancer screening programmes in that it specifically targets a high-risk population. So far, conducted European trials focused on current or former smokers with an age range between 50–74 years, a smoking history of 20–30 pack years and quit smoking within the last 10–15 years [4–7]. Two large randomised controlled trials, NLST (National Lung Screening Trial) and NELSON (Nederlands-Leuvens Longkanker Screenings Onderzoek), have demonstrated the benefits of LDCT lung cancer screening (LCS), and further European studies, such as the MILD (Multicentric Italian Lung Detection) and LUSI (Lung Tumour Screening and Intervention Trial) trials, also confirmed a reduction in lung cancer-specific mortality [4–7]. However, refinement of inclusion criteria for maximising the effectiveness of lung cancer screening may still be needed.

The current evidence has led the European Council to update its 2003 recommendation on cancer screening at the end of 2022, to include lung cancer among the cancers to be screened, and to encourage countries to study the feasibility and effectiveness of screening [8]. Several countries (e.g. Poland, Croatia, United Kingdom) already run, or are setting up, nationwide lung cancer screening programmes [9, 10]. Additionally, there are several lung cancer screening pilot projects in different European countries running or starting [11]. It is expected that governments and health care providers of other European countries will also approve similar initiatives. To foster the broad implementation of lung cancer screening in the member states of the European Union, the EU4Health programme has funded the SOLACE (strengthening the screening of lung cancer in Europe) project [12].

Early detection and appropriate management of pulmonary nodules are of paramount importance in LDCT LCS. Nodule management guidelines provide clinicians with a framework for risk stratification, follow-up, and appropriate diagnostic interventions.

It is essential to differentiate nodule management guidelines for incidental pulmonary nodules (e.g. Fleischner criteria [13]) from guidelines for screen-detected nodules (e.g. European Position Statement (EUPS) guidelines, the NELSON study nodule management protocol, the I-ELCAP (Early Lung Cancer Action Programme) recommendation and LungRADS v2022, which is the major US-American guideline) or guidelines for both screen-detected and incidental nodules (e.g. British Thoracic Society (BTS) guidelines) [14–17]. As there is no uniformity between them, we felt it important to summarise them and to propose a new approach (discussed in detail in the justification paper) aimed at minimising false positives, reassessment errors and the number of intermediate scans during screening, while also reducing overdiagnosis and the risk for stage-shift during follow-up.

To ensure standardisation, we also propose the use of structured reports, to enable consistent reporting of findings, improved communication, and reduced ambiguity.

## Nodule management guidelines for screen-detected pulmonary nodules

### Risk categories and nodule classification approaches

The rationale between different nodule management categories is an estimate of the risk of the nodule detected being a lung cancer. To date, it is accepted that risk can be summarised into three main categories

- Negative: estimated risk for lung cancer “very low”. The risk is low enough so that the time interval until the next annual screening round can be upheld. Some authors set the 1-year risk threshold at 1%, whereas others push up to 6% for the recommendation of annual screening rounds [18, 19]. This negative category represents up to 80–85% of all individuals undergoing LCS [5, 20].
- Indeterminate: estimated risk for lung cancer “low to intermediate”. The 1-year lung cancer risk for this category is heterogeneous across guidelines, with some guidelines using 2–3% risk as reference, while others pushing the boundary up to 10–30% [18, 19, 21]. The management of an indeterminate nodule requires a follow-up LDCT at an interval that is lower than the regular screening interval to ascertain nodule growth, size reduction or resolution. This follow-up LDCT is performed after a 1, 3, or 6-month interval, depending on the guideline. Indeterminate nodules are variably represented in the published series, found in 10–20% of subjects undergoing LCS.
- Positive: estimated risk for lung cancer “high”. In this case, diagnostic work-up and referral to a Multidisciplinary Team (MDT) meeting are needed to discuss management.

The existing guidelines notably diverge in their method for weighting the risk of a nodule being a lung cancer. This heterogeneity is associated with variable degrees of complexity of the classification system (Supplementary Material, Table S1).

Three main approaches for nodule classification can be summarised:

- (a) Morphological classification of nodule based on its detailed morphology with a series of specific combinations of morphological features that fit in a list of follow-up options. The most detailed example of such an approach is given by LungRADS v2022: this algorithm relies on fixed definitions that include nodules of different densities, pulmonary cysts, and airway nodules [20]. Under LungRADS v2022, the LCS reporting radiologist is called to identify all the features that apply and find the most “representative category” from among a list of about six major categories (e.g., from category 1 to category 4X).
- (b) Model-based classification of nodule risk by a mathematical model, e.g. a multivariable logistic regression model, that can be filled with a list of individual features and automatically provides a probability. The LCS reporting radiologist is called to enter each single feature into the model, which will automatically provide the estimate of lung cancer risk for that nodule. This automatically estimated risk is then assigned to a certain follow-up (or work-up) strategy. The example for this summarised format is found in the International Lung Screen Trial (ILST) and the British targeted lung health check (TLHC), both integrating the Brock model, in which the demographic, clinical, and imaging features are used for risk calculation. A definition for management of cystic nodules is missing in ILST and TLHC [10, 19].
- (c) Deep learning-based classification of nodule risk by a deep learning-based classification model. These models are typically fed with the image or a subimage around the nodule and optionally a few clinical parameters, and then typically process the image using deep convolutional neural networks to automatically compute a probability. No guidelines or management protocols have integrated these deep learning-based models yet, but several academic and commercial solutions exist, have been described in the literature [22, 23], and are being prospectively tested.

These approaches both have their pros and cons. Specifically, approach (a) will apply to most “common” nodules. Approaches (b) and (c) are likely to reduce the variability of interpretation, especially for complex nodules, because the radiologist is not required to search for the most “representative category,” but the risk categories are assessed by filling a risk model, either manually (b) or automatically (c). The ILST demonstrated that method (b) shows higher negative predictive value, allegedly related to additional information included in the model that does not relate to the nodule morphology but are still included in the calculation determining if the nodule is likely to be a cancer (e.g. age, family history of lung cancer, lobar location, number of nodules, presence of emphysema, etc) [24].

### Morphological criteria suggestive of malignancy

Current guidelines are based on density (solid vs subsolid), size and growth. However, there are several morphological criteria on CT that are suggestive, though not specific, for malignancy. Although these features are used in daily practice, publications document considerable inter-reader variability [2, 25]. This is especially true for patients with a high prevalence of pre-existing parenchymal changes (emphysema, fibrosis, silicosis, and bronchiectatic disease), where these morphological features become less specific, even though the underlying condition may increase the risk of developing lung cancer [26].

**Fig. 1:**
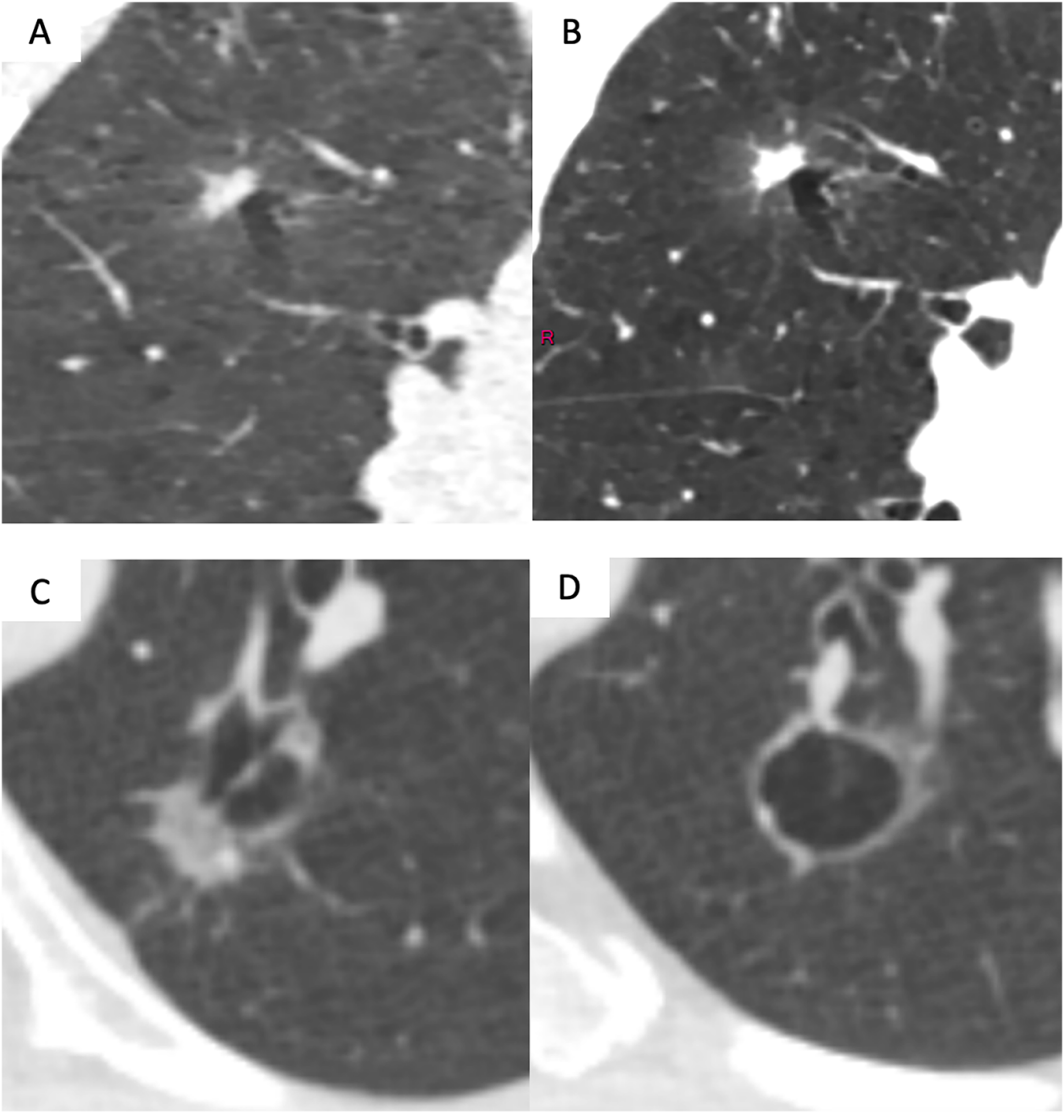
Suspicious morphological sign of ‘atypical cyst’ or ’cystic airspace’ in two patients. **A**, **B** Low-dose coronal CT images showing a 7-mm solid nodule in the wall of a right upper lobe cyst in a 60-year-old woman who smoked 31 pack-years, corresponding to an invasive adenocarcinoma staged pT1bN0R0. Standard-dose CT acquisition (**B**) performed preoperatively revealed a ground-glass component that was not detected on low-dose acquisition (**A**). **C**, **D** Low-dose axial CT images showing an atypical cyst of the left lower lobe in a 62-year-old woman with a smoking history of 40 pack-years. A solid part of 12 mm is extrinsically developed. A pT1b invasive adenocarcinoma was confirmed postoperatively

**Fig. 2:**
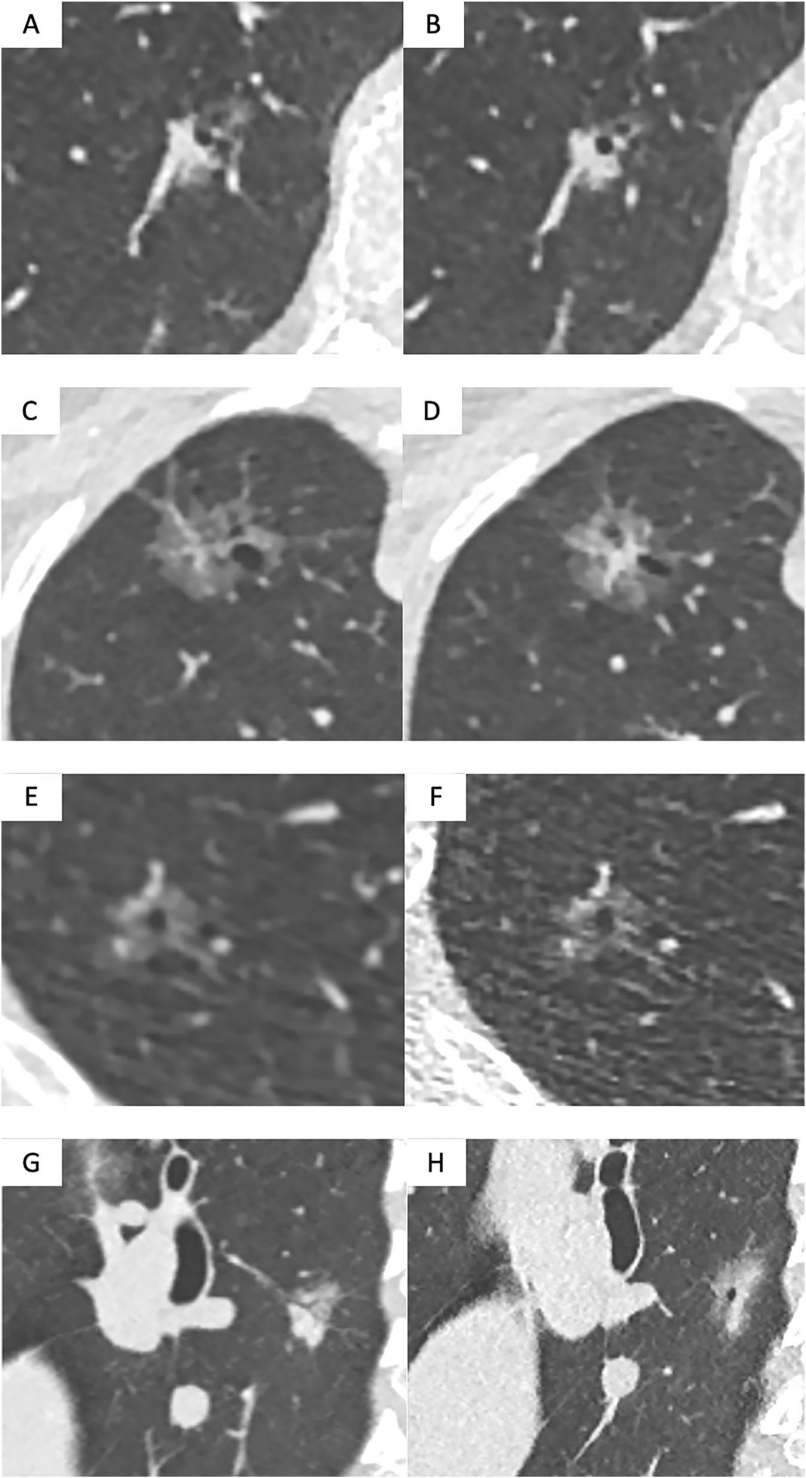
Suspicious morphological signs of ‘bubble-like lucencies’ and/or ‘air bronchogram’ in four patients. **A**, **B** Low-dose axial CT images showing a 17-mm part-solid nodule of the right lower lobe with an anterior ground-glass component (**A**) and bubble-like lucencies (**A**, **B**), in a 69-year-old woman who smoked 50 pack-years. Since the nodule has a solid component that is larger than 80% of the whole nodule, according to the ESTI guidelines, it would be managed as a solid nodule. A pT1bN0R0 invasive adenocarcinoma was confirmed postoperatively. **C**, **D** Low-dose axial CT images showing a part-solid nodule of the right upper lobe with a 7-mm solid component and bubble-like lucencies in a 64-year-old woman who smoked 35 pack-years. A pT1cN0R0 invasive adenocarcinoma was confirmed postoperatively. **E**, **F** Low-dose axial CT images in soft kernel (**E**) and high frequency kernel (**F**) showing a part-solid nodule of the right upper lobe with small non-measurable solid components and central bubble-like lucency in a 54-year-old woman with a smoking history of 35 pack-years. The high-frequency kernel image (**F**) is noisier. A pT1bN0R0 invasive adenocarcinoma was confirmed postoperatively. **G**, **H** Low-dose sagittal CT images showing a 24-mm part-solid nodule of the right lower lobe with an air bronchogram (**G**) and bubble-like lucency (**H**), in a 52-year-old woman with a smoking history of 30 pack-years. A pT1bN0R0 invasive adenocarcinoma was confirmed postoperatively

**Fig. 3:**
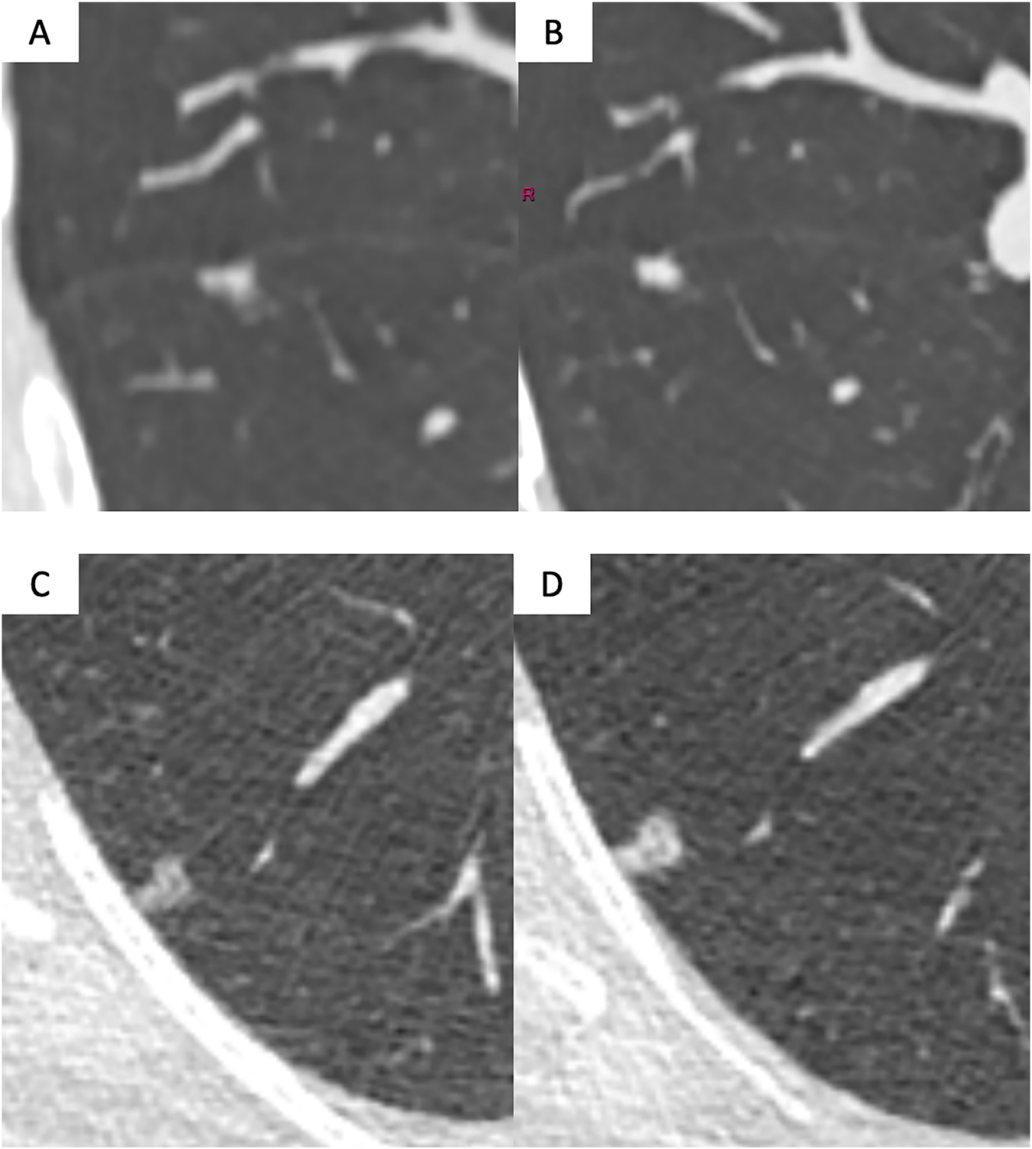
Suspicious morphological signs of ‘pleural indentation’ and ‘concave margin’ in two small invasive adenocarcinomas. **A**, **B** Low-dose axial CT images showing a part-solid nodule in the right lower lobe of a 57-year-old woman who had smoked 58 pack-years. Although still small, the nodule shows signs of pleural indentation. Histopathological examination confirmed the presence of an invasive adenocarcinoma with pleural invasion. The tumour was staged pT2N0R0. **C**, **D** Low-dose axial CT images showing a non-solid nodule of the right lower lobe in a 71-year-old man with a smoking history of 57 pack-years. The nodule shows a concave margin in the anterior aspect (**C**), then grows in size and density after 1 year (**D**)

Spiculation and signs of perinodular architectural distortion, e.g. fissure displacement, are known features suggesting that a solid nodule is malignant. Signs of perinodular distortion, such as pleural indentation, also apply for subsolid nodules (Figs. 1–3). Radio-pathological correlation has established that the size of a solid component is the best criterion to suggest invasiveness. A threshold of 5 mm for the solid component is generally accepted as a sign of invasiveness and corresponds to the pathological definition of a minimally invasive adenocarcinoma (MIA) in which the invasive portion should not exceed 5 mm [27]. However, it is also possible for invasive adenocarcinomas to take on the appearance of a pure ground glass nodule (GGN) [28]. If the solid component of a part-solid nodule is more than 80% of the entire nodule diameter, this nodule should be classified as a solid nodule [29].

Some subsolid nodules present with multiple solid components or solid components that are not accurately measurable. Generally, an increase in density over time—whether focal or diffuse—is indicative of malignancy. The concept of mass (nodule volume x nodule density) has been suggested to account for both the increase in size and density [30], but it is not applied in any of the current guidelines. Measurements of nodule mass require accurate segmentation of the whole nodule, including the subsolid component, which is not possible with all commercially available software today.

Other features indicative of an underlying malignancy have been proposed, including: dilated small airways, bubble-like lucencies, arc concave sign, sudden bronchus cut off, endoluminal filling of bronchi in follow-up, or narrowing of vessels [31, 32]. In addition to solid and subsolid nodule types, the “atypical pulmonary cyst” category has been included in the recently updated 2022 Lung-RADS version [20]. Cysts that develop irregular extrinsic or intrinsic nodular wall thickening over time are highly suspicious for malignancy.

Lung-RADS is the only management guideline that allows for upgrading nodules to a higher risk category (category 4X), if the CT shows morphological features that are considered suspicious. Although one study showed the positive effect of the 4X category on the correct estimation of risk, agreement between readers on the 4X category is moderate [33]. One possible explanation is the lack of an illustrated atlas accompanying the 4X category.

Examples of the various suspicious morphological signs are illustrated in Figs. 1–3.

### Morphological criteria for intrapulmonary lymph nodes and criteria suggesting benignity

Based on the results of the screening trials, the vast majority of small solid nodules are benign. A significant proportion of these are intrapulmonary lymph nodes (IPLNs), either located along a fissure (also termed PFO for perifissural opacity) or close to the costal pleura. This category must be recognised morphologically, as volumetric assessment of growth is not appropriate, since these lymph nodes can have growth rates in the order of those of malignant nodules, even though they are benign by definition [34]. Typical CT features of IPLNs are a noncalcified solid nodule with sharp margins; a round, oval, or polygonal shape; distanced 15 mm or less from the pleura and a diameter of 12 mm or less; and located below the level of the carina, since most cancer nodules misclassified as IPLNs are in the upper lobes [35]. Perifissural nodules represent up to 28% of nodules seen at CT screening for lung cancer [36]. The adoption of recommendations for perifissural nodules is variable. The BTS guideline recommends no further follow-up for nodules meeting morphologic criteria that are within 1 cm of a fissure or pleural surface and less than 10 mm [14]. With Lung-RADS v2022, juxtapleural nodules < 10 mm are downclassified to category 2. Unlike with BTS, there is no definition regarding maximum distance from the fissure. Other morphological features indicative of benignity include a well-defined smooth border, the presence of intranodular fat (attenuation ranging from −40 HU to −120 HU), and/or calcifications. Specific calcification patterns, such as central, diffuse, or ‘popcorn-like,’ are commonly associated with granulomas or hamartomas [31].

### Size categories and follow-up recommendations of the various guidelines

The method for measuring nodule size, either diameter or volume, varies between guidelines. Nodule volume, however, can be transformed into effective diameter by calculating the diameter of a perfect sphere of the same volume. Calculating volumes from diameters requires diameter measurements in three orthogonal planes but still is problematic if the nodule is irregular and deviates in shape from a perfect ellipsoid. In such cases with irregular shape-, the volume calculated from orthogonal diameters will overestimate the real volume.

Guidelines show some similarity regarding the size thresholds for solid nodules, with 6 mm defined as the lower threshold in Lung-RADS v2022, which roughly corresponds to the 100 mm^3^ proposed by the EUPS guideline [15]. The 15 mm upper threshold for a positive screen result according to the I-ELCAP corresponds to the Lung-RADS category 4B for referral to MDT discussion, whereas both the EUPS and BTS guidelines chose a threshold of 300 mm^3^ for solid nodules (effective diameter 8.3 mm), in line with the post-hoc analysis of the NELSON trial [37].

There are also differences with regard to which size of a pure GGN is considered a negative screening result: less than 8 mm according to NELSON, less than 3 cm according to LungRADS v2022 and whatever the size for pure GGNs, whether prevalent or incident, according to I-ELCAP [16, 17, 20]. Morphological nodule descriptors are consistent across various guidelines and are used not only to upstage nodules with malignant features but, more importantly, to downgrade larger nodules that exhibit benign morphology. Regarding the interval between screening rounds, annual rounds are the most widespread choice. The British TLHC and ILST are the only ones applying a biennial interval for their lowest risk category [10, 19].

In the various guidelines, the intervals for following indeterminate nodules vary between 1 month, 3 months and 6 months depending on the type of nodule. LungRADS v2022 follows pure GGNs larger than 3 cm after 6 months, while findings suggestive of an inflammatory or infectious process are reassessed after 1–3 months [20]. Nodules between 80 mm^3^ and 300 mm^3^ are reassessed at 3 months according to the BTS guideline [14]. If segmentation fails, an increase in mean diameter of > 1.5 mm (within a 12-month interval) with manual measurement results in a positive screen [20].

## Advantages and limitations of volumetric assessment of nodules

Based on the published evidence, computer-aided volumetric assessment of volume growth is clearly preferred to manual two-dimensional measurements for several reasons. For geometrical reasons, a doubling in volume (volume increase by 100%) only results in a 26% increase in diameter. It has been shown that manual measurements of diameter are subject to significant intra (±1.4 mm, 95% limits of agreement) and inter-observer (±1.7 mm, 95% limits of agreement) variability, which can lead to the mistaken belief that a nodule is growing when it is stable, and vice versa [38]. Some malignant nodules may show asymmetric growth patterns, mainly occurring along the z-axis, which will be identified by volumetry but not by two-dimensional diameter measurements [39].

Software-based volume measurements are an important improvement for the precision of nodule growth assessment, though several limitations need to be considered. Most systems use a combination of density thresholding and shape recognition. Volumetry software is less efficient for segmenting non-solid components with a small difference in attenuation from the surrounding normal lung [40]. In addition, cystic nodules or part-solid nodules pose a problem, even if segmentation is targeted towards only the solid component. Segmentation of solid nodules adjacent to the pleura or pulmonary vessels may overestimate nodule size due to the inclusion of neighbouring structures [41].

Acquisition and reconstruction parameters need to be kept as constant as possible during follow-up. To the extent that density thresholding is required, it is preferable to perform the volume analysis on standard reconstruction filters rather than on filters that emphasise spatial resolution [42]. Since the higher noise levels of hard kernels increase variability in volumetry, soft kernels are preferred to achieve more accurate and reproducible volumetric measurements of both solid and subsolid nodules. The radiation dose influences the noise level, which has an impact on the thresholding, even though iterative or deep learning-based reconstructions minimise the impact of low- or ultra-low-dose protocols on phantom studies [43–45].

Patient positioning on the CT table or in the degree of inspiration may induce variability. To quantify the impact of these patient-related factors, the same nodule was measured in two subsequent scans, with the patient getting on and off the table between scans. The relative difference between volumes strongly depended on segmentation accuracy; while the 95% confidence intervals for the difference between the two volume measurements were approximately ±12% for completely segmented nodules, this interval increased to approximately ±30% for incompletely segmented nodules. Inspiration level had a weak effect on measurement variability [46]. This experiment was carried out on only 20 patients with pulmonary metastases and has not been repeated with more recent segmenting software. Consequently, it is difficult to estimate current volumetric measurement variability.

Software from different vendors will provide different results [47]. Some allow segmentation errors to be corrected manually, while others do not. The same software should be used throughout a lung cancer screening programme, or at least as part of the follow-up of individual participants [48]. If new software is installed, earlier measurements have to be repeated if management may be affected.

## European Society of Thoracic Imaging (ESTI) proposal for management of nodules detected during lung screening

The rationale for developing and proposing these ESTI guidelines was that reviewing and amalgamating the current international guidelines would improve participant/patient care by putting a stronger focus on the aggressiveness of lesions. The rationale for the choices made is extensively discussed in a separate manuscript [49].

At baseline, aggressiveness is mainly derived from nodule type [50]. As previously discussed, a number of morphological features may also give an indication of malignancy. Showing examples of suspicious morphological signs (Figs. 1–3) is intended to improve inter-observer agreement.

Short-term follow-up is meant to identify fast-growing nodules before they undergo stage shift. Later follow-up is designed to identify slower-growing lesions.

Assessment of growth is key to assessing malignancy. Since most lung tumours exhibit exponential growth, volumetry with assessment of volume doubling time (VDT) can be used as a measure of growth rate [15, 51]. Growth can be estimated more accurately for shorter volume doubling times, longer follow-up intervals, and more precise volumetry. All nodule measurements are prone to measurement variability. In the case of volumetry, this depends on software, nodule morphology and—to a lesser degree—scanning technique [46]. To note is that growth is not a synonym of malignancy since benign nodules (e.g. hamartomas) can exhibit slow growth patterns with VDTs comparable to some slow-growing malignant nodules. Manual measurement with a size threshold of >1.5 mm as a criterion for substantial growth is recommended for the solid component of subsolid nodules, since volume segmentation often is not sufficiently accurate in this setting. Manual measurement of the solid component should be performed on lung window settings. For manual measurements of the solid component in part-solid nodules, the use of a hard kernel might be preferred to more easily distinguish the boundaries of the solid component because of improved edge definition.

**Fig. 4:**
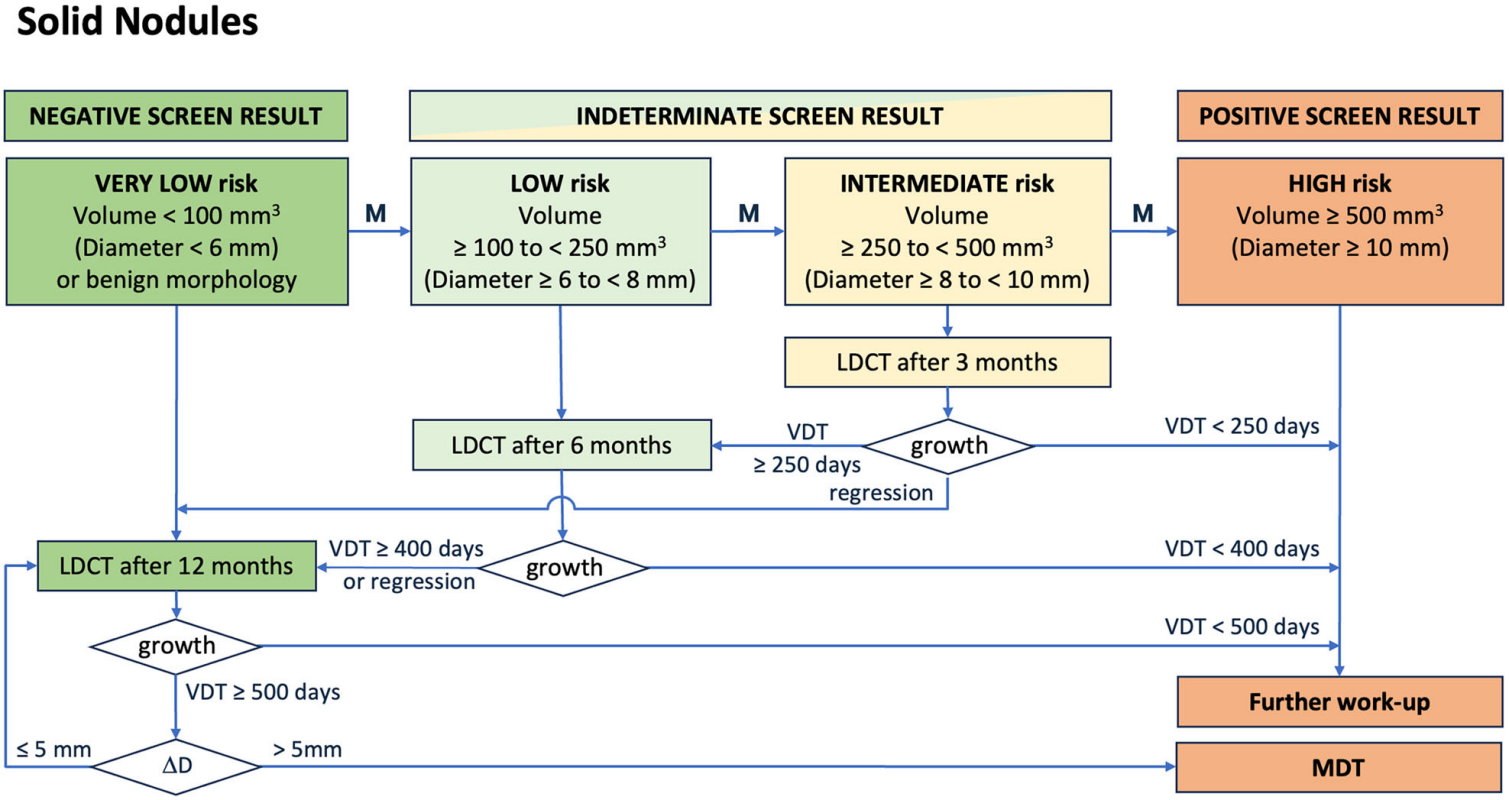
Flowchart for management of solid nodules detected at baseline. M = suspicious morphology upgrades risk to next category: spiculation, architectural distortion (pleural tag, fissural displacement), cystic component, bubble-like lucencies, concave sign, narrowed vessels. Benign morphology: calcification (central, diffuse, popcorn-like), fat components, typical intrapulmonary lymph node morphology (smooth margins, oval, lentiform, or triangular shape, < 1 cm, distance to pleura < 1 cm, under the carina). Growth *=* substantial growth, defined as follows: • If volumetry is possible: VDT < 250 days at 3 months, VDT < 400 days at 6 months and VDT < 500 days at ≥ 12 months. • If volumetry fails: visually verifiable increase in average diameter of > 1.5 mm over a time interval of maximally 1 year, or substantial change in morphology. A decrease in size may indicate a benign process (inflammation, infection, other) and prompts ongoing follow-up to ensure shrinkage continues. Δ*D* = change in effective diameter relative to baseline, derived from volume or from manual measurements if volumetry fails. MDT ***=*** multidisciplinary team decision is advised if the effective diameter of a slow-growing nodule increases by more than 5 mm from baseline

**Fig. 5:**
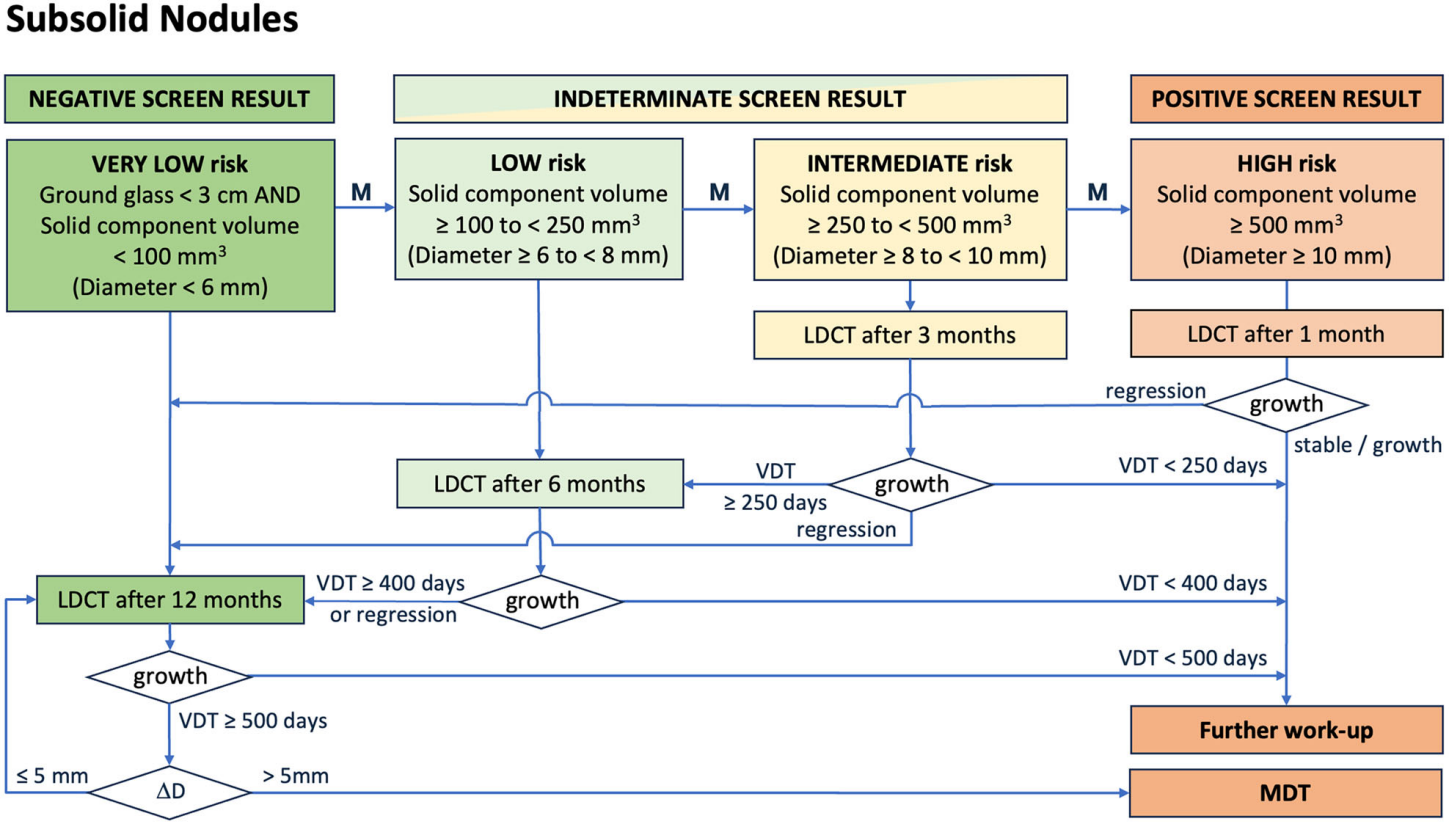
Flowchart for management of subsolid nodules detected at baseline. M* =* suspicious morphology upgrades risk to next category: spiculation, architectural distortion (pleural tag, fissure displacement), cystic component, bubble-like lucencies, concave sign, bronchus cut-off, ground glass component ≥ 3 cm in average or effective diameter. Solid component: if the solid component of a part-solid nodule is more than 80% of the entire nodule diameter, this nodule should be classified as a solid nodule. Growth = substantial growth, defined as follows: • If volumetry is possible: VDT < 250 days at 3 months, VDT < 400 days at 6 months and VDT < 500 days at ≥ 12 months. • If volumetry fails: visually verifiable increase in average diameter of > 1.5 mm over a time interval of maximally 1 year, or substantial change in morphology. Regression = complete disappearance or marked decrease in size, density or volume of subsolid nodules. Δ*D* = change in effective diameter relative to baseline, derived from volume or from manual measurements if volumetry fails. MDT ***=*** multidisciplinary team decision is advised if the effective diameter of a slow-growing nodule increases by more than 5 mm from baseline

**Fig. 6:**
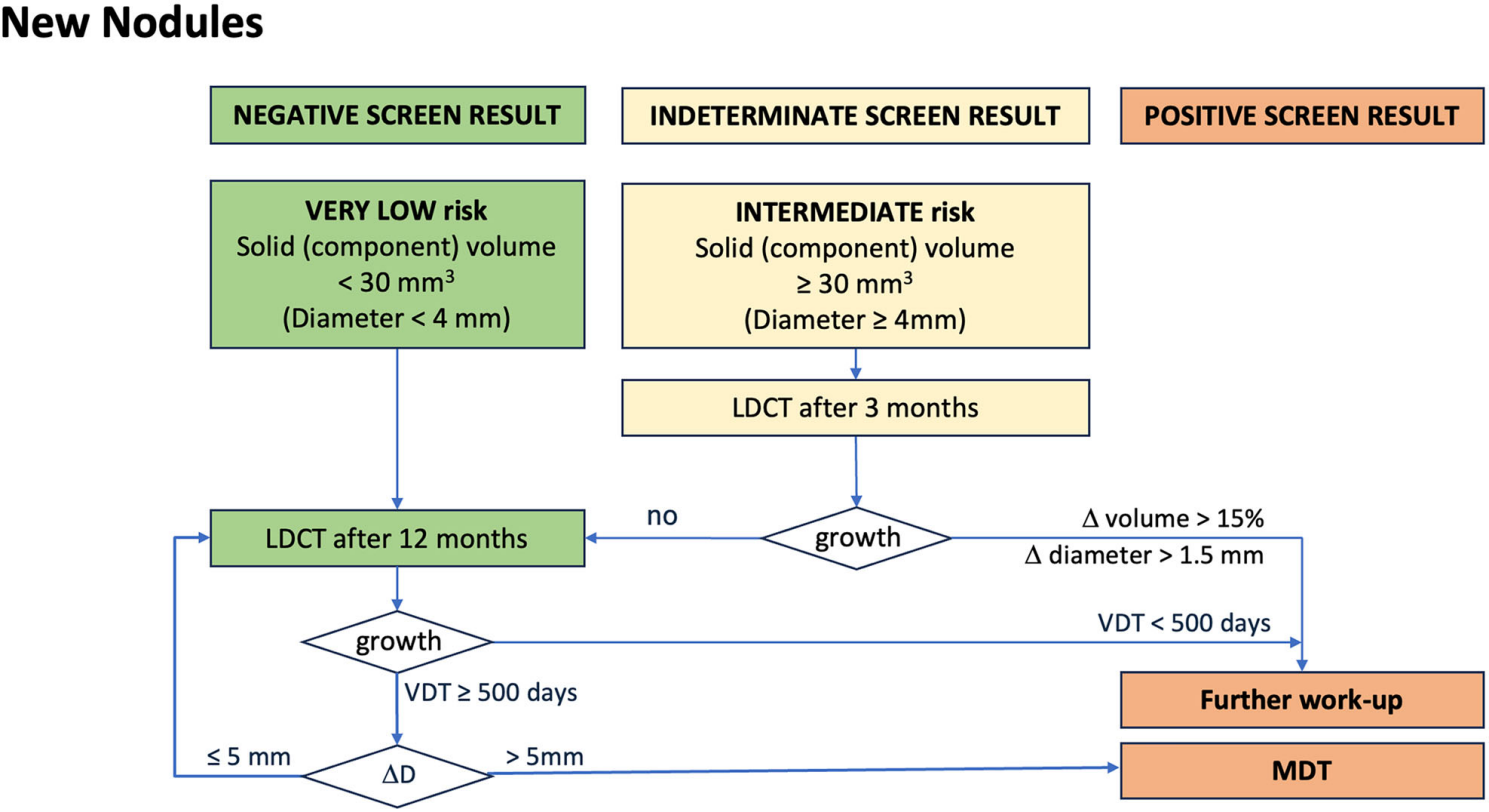
Flowchart for management of new nodules. New nodules that had been missed or not reported on previous scans are managed according to the same rules as nodules found at baseline

The proposal for nodule management of solid and subsolid nodules, both at baseline, during follow-up and when new, is summarised in Figs. 4–6 and Table 1. Annual screening should continue until the participant would no longer benefit from the early detection of lung cancer (e.g. they develop a life-limiting comorbidity) or until screening is stopped in accordance with programme-specific protocols (e.g. an upper age limit).

**Table 1:** Overview of management of solid, subsolid and new nodules

Baseline	Morphology	Density	Criteria	Management
Negative	No nodule			12 months
	Benign features	Any	Benign calcification, fat components, typical intrapulmonary lymph node or infection	
	No suspicious features	Solid (component)	< 100 mm^3^ (< 6 mm)	
		Non-solid (component)	< 30 mm	
Indeterminate	Suspicious features	Solid (component)	< 100 mm^3^ (< 6 mm)	6 months
	No suspicious features		≥ 100 to < 250 mm^3^ (≥ 6 to < 8 mm)	
		Non-solid (component)	≥ 30 mm	
	Suspicious features	Solid (component)	≥ 100 to < 250 mm^3^ (≥ 6 to < 8 mm)	3 months
	No suspicious features		≥ 250 to < 500 mm^3^ (≥ 8 to < 10 mm)	
Positive	No suspicious features	Part-solid	≥ 500 mm^3^ (≥ 10 mm)	1 month
		Solid	≥ 500 mm^3^ (≥ 10 mm)	Work up
	Suspicious features	Solid (component)	≥ 250 mm^3^ (≥ 8 mm)	
**Follow-up**	**Prevalent/new**	**Density**	**Criteria**	**Management**
Negative	Prevalent nodule	Solid (component)	Regression OR VDT ≥ 400 d after 6 months OR VDT ≥ 500 d after 12 months average diameter increase ≤ 1.5 mm/year^1^	12 months
		Non-solid (component)	Size remains < 30 mm	
	New nodule	Solid (component)	< 30 mm^3^ (< 4 mm)	
		Non-solid (component)	Any	
Indeterminate	Prevalent nodule	Solid (component)	VDT ≥ 250 d after 3 months average diameter increase ≤ 1.5 mm/3 months^1^	6 months
		Non-solid (component)	Size increases to ≥ 30 mm	
	New nodule	Solid (component)	≥ 30 mm^3^ (≥ 4 mm)	3 months
Positive	Prevalent nodule	Part-solid	Persistent solid component ≥ 500 mm^3^ (≥ 10 mm)	MDT
		Solid	Total diameter growth > 5 mm	
		Non-solid	Development of solid core	
	Prevalent nodule	Solid (component)	VDT < 250 days after 3 months OR VDT < 400 days after 6 months OR VDT < 500 days after 12 months average diameter increase > 1.5 mm/year^1^	Workup
	New nodule follow-up	Solid (component)	> 15% volume growth at 3 months > 1.5 mm diameter growth at 3 months	

^1^If volumetry fails: visually verifiable increase in average diameter of > 1.5 mm over a time interval of maximally 1 year, or substantial change in morphology

A structured report template is presented in the supplementary material.

## Future perspectives

Computer-aided detection tools and artificial intelligence-based algorithms for lung nodule detection and volumetric assessment have demonstrated promising performance, although based on retrospective studies [52–54]. More than fifteen CE-certified algorithms are now available in Europe for clinical use. However, there is no systematic reimbursement in the majority of European countries. Moreover, recent studies have shown that deep learning-based models using image data alone are able to outperform multivariable risk models such as Brock for malignancy risk estimation [22, 23]. As a result, when prospectively validated, these models may in the future be used to guide nodule management, resulting in faster recognition of malignant nodules and fewer follow-up CT scans for benign nodules [55]. Radiomics and deep learning approaches have been developed to distinguish between pre-invasive and invasive forms of adenocarcinoma presenting as subsolid nodules. However, their performance is not yet superior to that of the radiologists’ measurements of the size of the solid component [56].

There is sufficient evidence showing that screening for lung cancer using low-dose CT in a high-risk population of current and former smokers reduces lung cancer mortality. Smoking, however, is not only a major risk factor for lung cancer but also for COPD and cardiovascular disease. All three (“BIG 3”) account for most of the deaths in screening participants and can be detected and quantitatively assessed with non-contrast, ungated chest CT [57]. This might open the way to further reduction of overall mortality in screening participants, but appropriate guidelines for quantification and further management remain to be defined. Future LDCT screening programmes, therefore, should address these issues adequately. Alternatives to LDCT for lung cancer screening are sparse. Despite technical feasibility, satisfactory nodule detection rates and even estimated cost-effectiveness [58], lung MRI requires specific expertise and dedicated MR scan time, both of which are not available on a sufficiently large scale [59]. A broad application beyond individual cases is therefore not expected in the near future. There is extensive research on the use of blood- and breath biomarkers to help determine who should be screened or to help determine if a nodule detected by LDCT is malignant. While such blood markers hold substantial promise, cost-effectiveness is not yet established, and sufficient data from large-scale clinical validation is still lacking.

## Conclusion

While scientific evidence confirms the benefits of LCS, the next challenge will be the effective implementation of suitable programmes, minimising the harms and maximising the benefits of LDCT LCS. The proposed ESTI/European Society of Radiology nodule management concept refines the definition of positive, indeterminate, and negative screen results and will contribute to controlling false positives, reassessment errors, and the number of intermediate CT scans while also reducing overdiagnosis and the risk for stage-shift during follow-up. Ongoing implementation trials will offer the opportunity to further validate and refine this approach and prospectively assess the impact of artificial intelligence in LCS.

## Supplementary information

ELECTRONIC SUPPLEMENTARY MATERIAL

Table S1

## Abbreviations

BTS: British Thoracic Society
CT: Computed tomography
ESTI: European Society of Thoracic Imaging
EUPS: European Position Statement
GGN: Ground glass nodule
I-ELCAP: International Early Lung Cancer Action Program
ILST: International lung screen trial
LDCT: Low-dose computed tomography
MDT: Multidisciplinary team
NELSON: Nederlands–Leuvens Longkanker Screenings Onderzoek
TLHC: Targeted lung health check
VDT: Volume-doubling time
