# Supplementary material for: Lung cancer screening with low-dose CT: definition of positive, indeterminate, and negative screen results. A nodule management recommendation from the European Society of Thoracic Imaging
Source: Eur Radiol. 2025 Jul 1;36(1):135–47. doi: 10.1007/s00330-025-11648-4 (PMC12711968; doi:10.1007/s00330-025-11648-4)
Supplement: Supplementary file 1 — ELECTRONIC SUPPLEMENTARY MATERIAL [file 330_2025_11648_MOESM1_ESM.pdf]

Lung Cancer Screening with low-dose CT: Definition of positive, indeterminate, and negative screen results. A Nodule Management Recommendation from the European Society of Thoracic Imaging

ELECTRONIC SUPPLEMENTARY MATERIAL

Eur Radiol (2025) Snoeckx A, Silva M, Prosch H, et al.

ESTI STRUCTURED REPORT TEMPLATE
Low-Dose CT screening (1/2)

Patient and examination data

Name: ____________________ First name: ____________________

Date of birth: ____________________

Date of previous CT: ____________________ Date of current CT: ____________________

DLP (mGy.cm) : ____________________ CTDI vol (mGy): ____________________

POSITIVE SCREENING RESULT

- ☐ **Solid nodule** with volume ≥ **500 mm³** (diameter ≥ 10 mm)
- ☐ **Solid nodule** with volume ≥250 to < 500 mm³ (diameter ≥ 8 to < 10 mm) and **suspicious morphology** (*)
- ☐ Subsolid **pure ground glass nodule ≥ 3 cm** in average or effective diameter
- ☐ Subsolid **nodule** with solid component volume ≥ **500 mm³** (≥ 10 mm) OR **ground glass component > 3 cm** → do FU LDCT after 1 month

  No regression: referral MDT

  Regression: FU LDCT after 12 months
- ☐ **Solid and subsolid nodules at follow-up with substantial growth**

  If volumetry is possible: VDT < 250 days at 3 months, VDT < 400 days at 6 months and VDT < 500 days at ≥ 12 months

  If volumetry fails: visually verifiable increase in size of > 1.5 mm over a time interval of maximally 1 year, or substantial change in morphology
- ☐ Truly new nodules with volume ≥ **30 mm³** (≥ 4 mm) with growth after 3 months follow-up
- ☐ Slow-growing nodules with increase by more than 5 mm from baseline: refer to MDT

* Suspicious morphology: spiculation, pleural indentation, cystic component, bubble-like lucencies, concave sign, bronchus cut-off

INDETERMINATE SCREENING RESULT

- ☐ **Solid nodule** volume ≥ **100 mm^3 and < 250 mm^3** (diameter ≥ 6 mm to < 8 mm)
  - No suspicious morphology: FU LDCT after 6 months
  - Suspicious morphology: FU LDCT after 3 months
- ☐ **Solid nodule** volume **≥250 mm^3 and < 500 mm^3** (diameter ≥ 8 mm to < 10 mm)
  - No suspicious morphology: FU LDCT after 3 months
  - Suspicious morphology: becomes positive screen result
- ☐ **Part-solid nodule** with solid component volume **<100 mm^3** (< 6 mm) and total size <3 cm AND suspicious morphology (*)
- ☐ **Part-solid** nodule with solid component volume **≥ 100 mm^3 and < 250 mm^3** (diameter ≥ 6 mm to < 8 mm)
  - No suspicious morphology: FU LDCT after 6 months
  - Suspicious morphology: FU LDCT after 3 months
- ☐ Part-**solid nodule** with solid component volume **≥250 mm^3 and <500 mm^3**
  - No suspicious morphology: FU LDCT after 3 months
  - Suspicious morphology: becomes positive screen result
- ☐ Focal consolidation of **infectious appearance**: FU 3 months post antibiotics
- ☐ Bulging or obstructing **endoluminal lesions** (other than mucous) in subsegmental or smaller bronchi: FU LDCT after 6 months
- ☐ **Focal endotracheal and proximal endobronchial** abnormalities (other than mucous): FU LDCT after 3 months

* Suspicious morphology: spiculation, pleural indentation, cystic component, bubble-like lucencies, concave sign, bronchus cut-off

NEGATIVE SCREENING RESULT

- ☐ No nodule
- ☐ **Solid nodules** with volume **< 100 mm^3** (diameter < 6mm)
- ☐ Subsolid **pure ground glass** nodule **< 3cm**
- ☐ Subsolid **part-solid nodule** with **solid component < 6mm** and **total size <3cm** (without morphological criteria suggesting malignancy)
- ☐ Nodule with **typically benign nodule characteristics** (intranodular fat or intrapulmonary lymph node criteria*)

* Criteria for intrapulmonary lymph node: typical morphology with smooth margins, oval, lentiform or triangular shape, < 1cm, distance to pleura < 1 cm, under the carina
